# Successful Repeated Thrombolysis for Obstructed Mechanical Mitral Valve Prosthesis: A Case Report From a Resource-Limited Setting

**DOI:** 10.7759/cureus.100910

**Published:** 2026-01-06

**Authors:** Milan Mehta, Nileshkumar Khanpara, Jayvirsinh Atodariya

**Affiliations:** 1 Cardiology, Sardar Patel Hospital and Heart Institute, Bharuch, IND; 2 General Medicine, Om Hospital and ICU, Surat, IND

**Keywords:** anticoagulation adherence, mechanical mitral valve, prosthetic valve thrombosis, recurrent valve thrombosis, streptokinase, subtherapeutic anticoagulation, thrombolytic therapy

## Abstract

We report the case of a 40-year-old man with rheumatic heart disease (a condition where the cardiac valve is damaged due to prior streptococcal infection). He previously underwent mechanical mitral valve replacement with a bileaflet prosthesis and later developed recurrent episodes of acute obstructive prosthetic valve thrombosis. He presented first in 2023 and again in 2025 with rapidly progressive dyspnea, orthopnea, diminished mechanical valve sounds, and signs of hemodynamic compromise. Both events followed prolonged periods of inconsistent warfarin use and inadequate international normalized ratio (INR) monitoring related to financial limitations and restricted access to outpatient care. Transthoracic echocardiography during each admission demonstrated markedly elevated transmitral gradients and restricted leaflet motion, confirming obstructive thrombosis of the mechanical mitral valve.

Because urgent surgical intervention was unavailable and transfer delays carried additional risk, each episode was managed with low-dose, slow-infusion streptokinase after multidisciplinary evaluation and informed consent. The patient showed prompt and sustained improvement during both thrombolytic courses, with resolution of respiratory distress, normalization of hemodynamics, and restoration of prosthetic leaflet mobility. Follow-up echocardiography confirmed thrombus resolution and a return to acceptable transvalvular gradients. No major bleeding, systemic embolization, or neurological complications occurred during either hospitalization. He was transitioned back to therapeutic warfarin levels and discharged in stable condition with reinforced counseling regarding anticoagulation adherence.

This case demonstrates the successful use of repeated thrombolytic therapy for recurrent obstructive mechanical mitral valve thrombosis when surgical management is inaccessible. The consistent clinical and echocardiographic responses across two separate episodes emphasize the importance of early recognition, appropriate patient selection, and close monitoring during thrombolytic treatment. The case also highlights persistent challenges in maintaining reliable anticoagulation in resource-limited environments, where consistent INR monitoring and continuity of care may be difficult to achieve.

## Introduction

Prosthetic valve thrombosis (PVT) is defined as thrombus formation on or near a prosthetic heart valve, leading to impaired valve function. PVT can be classified as obstructive (causing significant hemodynamic compromise due to impaired leaflet motion) or non-obstructive (often asymptomatic or causing only mild dysfunction). PVT represents one of the most serious complications of mechanical heart valve implantation, with an estimated incidence ranging from 0.3% to 1.3% per patient-year depending on valve type, anatomical position, and adequacy of anticoagulation control [1,2]. Despite advances in valve design, anticoagulation strategies, and clinical management, PVT continues to pose significant challenges to clinicians and remains associated with substantial morbidity and mortality. Left-sided prostheses, particularly those in the mitral position, are disproportionately affected, accounting for approximately 60-70% of all PVT cases, primarily due to lower transvalvular flow velocities and larger valve surface area compared to the aortic position, which create favorable conditions for thrombus formation [1,2].

Among the multiple recognized risk factors contributing to PVT development, subtherapeutic anticoagulation remains one of the most important and modifiable determinants, identified in up to 70-80% of cases [2,3]. The clinical presentation of PVT spans a broad spectrum, ranging from asymptomatic valve dysfunction detected incidentally on routine echocardiography to fulminant cardiogenic shock and sudden cardiac death, depending on the degree of valve obstruction, the rapidity of thrombus formation, and the presence of systemic embolization [3,4]. Obstructive PVT, characterized by impaired leaflet motion and hemodynamic obstruction as outlined above, constitutes a true medical and surgical emergency, frequently associated with hemodynamic compromise, heart failure, systemic embolization, and high mortality rates ranging from 10-40% depending on clinical severity and treatment approach [1,2,4,5].

Contemporary international guidelines, including the 2017 European Society of Cardiology/European Association for Cardio-Thoracic Surgery (ESC/EACTS) guidelines and the 2020 American College of Cardiology/American Heart Association (ACC/AHA) guidelines, recommend urgent surgical intervention, typically redo valve replacement or thrombectomy, as the preferred first-line treatment strategy for obstructive left-sided PVT, particularly for critically ill patients (New York Heart Association, NYHA, class III-IV symptoms) or those with large thrombus burden [1,3,4]. Surgery, however, carries substantial perioperative risks, with reported mortality rates of 10-15% for elective reoperations and up to 30-40% in emergency settings, influenced by patient clinical status, comorbidities, and institutional surgical expertise [5,6].

Thrombolytic therapy is generally reserved for patients deemed unsuitable for surgery due to prohibitive surgical risk, those with small thrombus burden, right-sided PVT, or in circumstances where surgical facilities are unavailable, or access is significantly delayed [1,3,5]. Evidence from multiple observational studies and meta-analyses has demonstrated that thrombolytic agents, including streptokinase, urokinase, recombinant tissue plasminogen activator (rtPA), and tenecteplase, can achieve success rates of 70-90% in resolving obstructive PVT, defined as complete restoration of normal valve function and thrombus resolution [7-12]. Nonetheless, thrombolysis carries inherent risks, most notably systemic embolization and major bleeding [5,6,13,14]. To mitigate these complications, recent protocols employing low-dose, slow-infusion regimens have shown promise in improving safety profiles while maintaining therapeutic efficacy [7,11,12].

Despite these advances, the literature remains sparse regarding the feasibility, safety, and outcomes of repeated thrombolytic therapy in patients experiencing recurrent obstructive mechanical valve thrombosis. Most published reports describe single episodes of PVT treated with thrombolysis, with relatively few cases documenting successful repeated treatment in the same patient [15,16]. Additionally, real-world management of PVT in low-resource settings is frequently constrained by limited access to cardiac surgery, prolonged referral delays, financial barriers, and inadequate anticoagulation monitoring infrastructure, necessitating alternative therapeutic approaches and guideline adaptation to local contexts [17,18].

We describe a case of obstructive mechanical mitral valve thrombosis successfully treated twice with low-dose, slow-infusion streptokinase in a resource-limited setting where emergent cardiac surgery was unavailable. This case provides valuable insights into the management of recurrent PVT in resource-constrained environments, demonstrating the feasibility and effectiveness of repeated thrombolytic therapy as a life-saving alternative, while highlighting the persistent challenges of anticoagulation management and the critical importance of patient education and follow-up systems in preventing recurrence.

## Case presentation

Patient background and surgical history

A 40-year-old male patient with a documented background of rheumatic heart disease (RHD) underwent mechanical mitral valve replacement with a 29 mm St. Jude Medical bileaflet prosthesis in 2020. A bileaflet prosthesis is a type of mechanical valve with two semicircular leaflets that open and close to regulate blood flow. His pre-operative evaluation revealed severe mitral stenosis with preserved left ventricular systolic function. The perioperative course was uneventful, and he was discharged on lifelong warfarin therapy with a target international normalized ratio (INR) of 2.5-3.5, which is the recommended therapeutic range for mechanical mitral valve prostheses. However, his long-term follow-up was inconsistent due to significant financial constraints and logistical barriers to healthcare access, resulting in irregular INR monitoring and variable adherence to prescribed anticoagulation therapy, a pattern unfortunately common in resource-limited settings.

First episode of prosthetic valve thrombosis in 2023

Approximately three years after valve replacement, in 2023, the patient presented to the emergency department with a five-day history of progressive exertional dyspnea that rapidly evolved to dyspnea at rest, accompanied by orthopnea requiring elevation with multiple pillows, paroxysmal nocturnal dyspnea, and progressive fatigue. He denied chest pain, syncope, or fever but admitted to irregular warfarin use over the preceding months, accompanied by irregular INR monitoring in his rural residential area. He also reported subjective perception of decreased intensity of his mechanical valve sounds, a concerning symptom suggesting valve dysfunction.

Physical examination revealed a patient in moderate-to-severe respiratory distress with tachypnea (respiratory rate 26 breaths/minute), sinus tachycardia (heart rate 110 beats/minute), blood pressure 96/60 mmHg, and oxygen saturation 92% on room air. Jugular venous pressure was mildly elevated at approximately 8-10 cm H₂O. Cardiovascular examination demonstrated a markedly diminished mechanical valve closing click, a critical finding suggesting restricted leaflet motion and a grade 3/6 holosystolic murmur radiating to the axilla, consistent with mitral regurgitation. Respiratory examination revealed bibasilar inspiratory crackles extending to the mid-lung fields, indicating pulmonary congestion. Bilateral lower extremity examination showed mild pitting edema up to the ankles.

Laboratory evaluation revealed subtherapeutic anticoagulation with an INR value of 1.3, well below the therapeutic target of 2.5-3.5. Creatine kinase-myocardial band (CK-MB) was 18 U/L (normal range: 0-25 U/L), and troponin-I was 0.012 ng/mL (normal range: <0.04 ng/mL), ruling out acute myocardial ischemia. The complete blood count was within normal limits, including a hemoglobin of 14.2 g/dL (normal range: 13.5-17.5 g/dL), white blood cell count of 6.4 ×10⁹/L (normal range: 4.0-11.0 ×10⁹/L), and platelet count of 250 ×10⁹/L (normal range: 150-450 ×10⁹/L). Renal function tests were unremarkable, with a serum creatinine of 0.9 mg/dL (normal range: 0.6-1.3 mg/dL) and blood urea nitrogen of 14 mg/dL (normal range: 7-20 mg/dL). Liver function tests were also normal, including aspartate aminotransferase (AST) 22 U/L (normal range: 10-40 U/L), alanine aminotransferase (ALT) 24 U/L (normal range: 7-56 U/L), and total bilirubin 0.8 mg/dL (normal range: 0.2-1.2 mg/dL). Inflammatory markers were within normal limits, with an erythrocyte sedimentation rate (ESR) of 10 mm/hr (normal: <20 mm/hr) and a C-reactive protein (CRP) of 1.2 mg/L (normal range: <5 mg/L). Serum electrolytes were also normal, including sodium 140 mmol/L (normal range: 135-145 mmol/L), potassium 4.2 mmol/L (normal range: 3.5-5.0 mmol/L), and chloride 102 mmol/L (normal range: 98-107 mmol/L). Electrocardiography demonstrated sinus tachycardia without acute ischemic changes. Chest radiography showed cardiomegaly with prominent pulmonary vascular markings consistent with pulmonary venous congestion.

**Figure 1:**
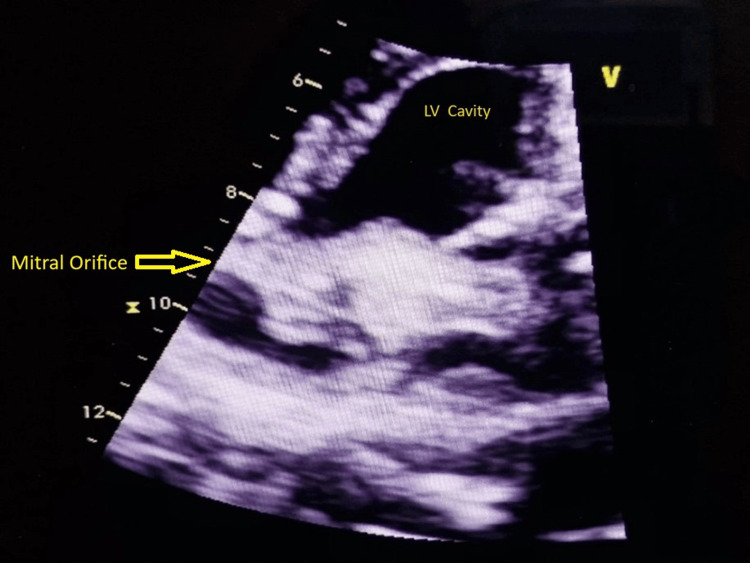
Echocardiography showing obstructive thrombus on mechanical mitral valve in PSAX at mitral level during first presentation PSAX - parasternal short axis view; LV - left ventricle

**Figure 2:**
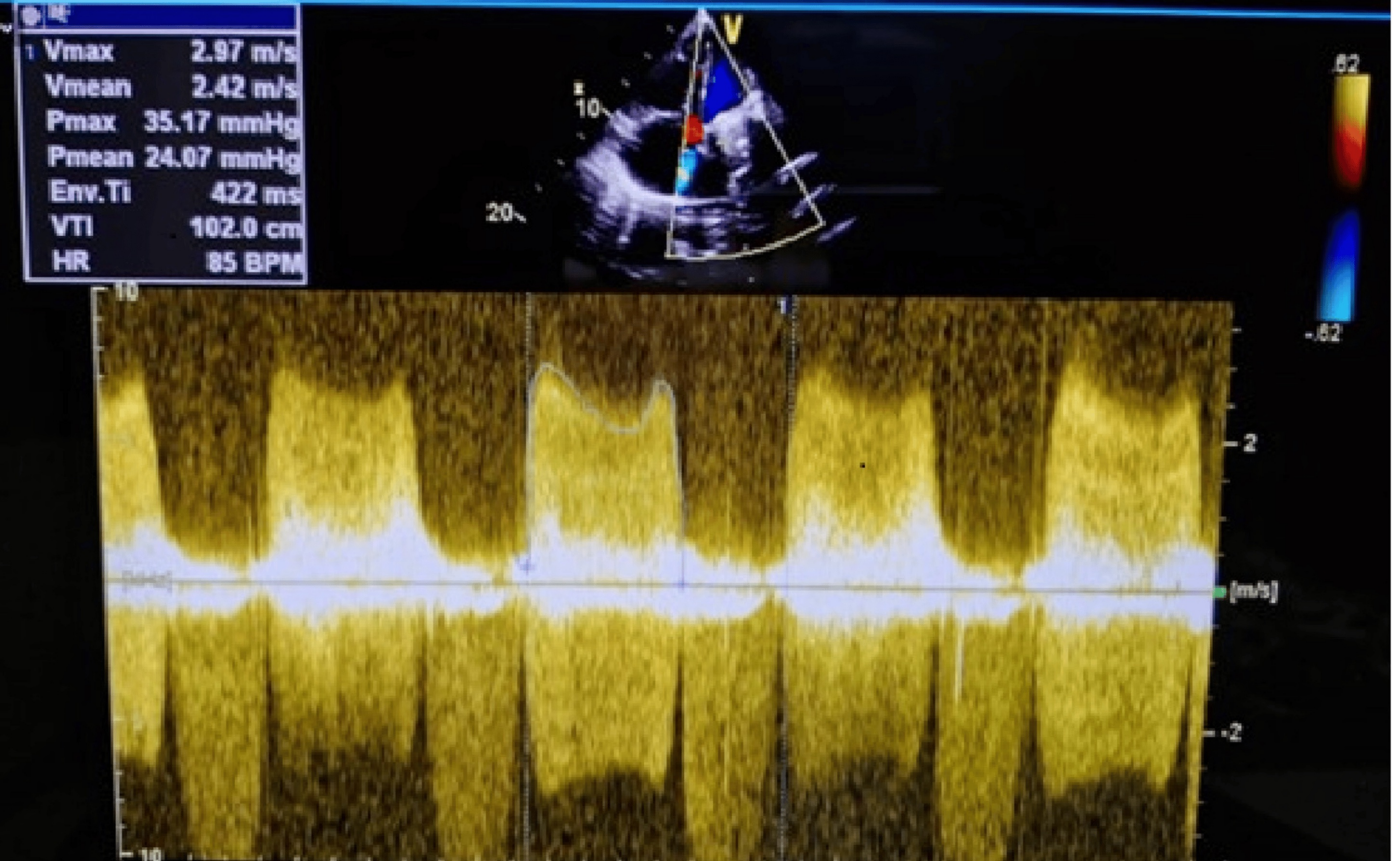
Continuous wave Doppler ultrasound during first presentation Continuous wave Doppler showing severely elevated transmitral gradients (Pmax 35.17 mmHg, Pmean 24.07 mmHg), indicating obstruction of the prosthetic valve. Pmax - peak gradient; Pmean - mean gradient

Urgent transthoracic echocardiography (TTE) demonstrated markedly elevated transmitral gradients with peak gradient of 35 mmHg and mean gradient of 24 mmHg (normal mechanical mitral valve mean gradient <5 mmHg), significantly restricted leaflet motion with reduced excursion and echodense material measuring approximately 1.2×0.8 cm attached to the atrial surface of the prosthetic valve, suggestive of thrombus on prosthetic valve causing significant obstruction (Figures 1-2). Left ventricular systolic function was preserved with an ejection fraction of 55% and the left atrium was moderately dilated. 

Given the critical clinical status with NYHA class IV symptoms, hemodynamic compromise, and obstructive PVT, the case was urgently discussed in a multidisciplinary team meeting involving cardiology, critical care medicine, and internal medicine specialists. Emergency cardiothoracic surgery was deemed the optimal treatment per guidelines; however, surgical intervention was unavailable at our institution due to a lack of on-site cardiac surgical facilities and qualified cardiothoracic surgeons. Referring to the nearest cardiac surgery center would have caused an estimated minimum delay of 48-72 hours for an emergency procedure. Given the patient's deteriorating hemodynamic status, prohibitive risk of further deterioration during transfer, and the immediate life-threatening nature of the condition, the multidisciplinary team determined that thrombolytic therapy represented the only feasible life-saving option.

Following a comprehensive discussion with the patient and his family regarding the potential risks of thrombolytic therapy, including stroke, systemic embolization, major bleeding, treatment failure necessitating emergency surgery, and the possibility of death, as well as the risks of conservative management or delayed surgical referral, informed written consent was obtained. The patient and family expressed a clear preference for immediate intervention given the severity of symptoms and accepted understanding of the associated risks.

Although transesophageal echocardiography (TEE) is considered superior to TTE for detailed assessment of prosthetic valve thrombosis, TEE was not performed during either presentation. In both episodes, the combination of classical clinical features (acute dyspnea, diminished mechanical valve clicks, and hemodynamic compromise), markedly elevated transvalvular gradients on TTE, and visible echodense masses on the prosthetic valve provided sufficient diagnostic confidence to proceed with urgent therapy. Given the patient's unstable clinical condition and the immediate life-threatening nature of obstructive prosthetic valve thrombosis, delaying treatment to plan and perform TEE was deemed likely to result in loss of precious time without altering management.

First thrombolysis course and outcome

The patient was transferred to the intensive care unit (ICU) for close hemodynamic and neurological monitoring. Intravenous streptokinase was administered according to a standardized institutional protocol for PVT: initial loading dose of 250,000 IU administered over 30 minutes, followed by continuous infusion at 100,000 IU/hour for 24 hours (total dose 2,650,000 IU). This low-dose, slow-infusion regimen was selected based on contemporary evidence suggesting an improved safety profile compared to traditional higher-dose protocols. Concomitant supportive therapies included supplemental oxygen, diuretics for volume management, and close monitoring with continuous telemetry, pulse oximetry, and continuous observation in the intensive care unit.

**Figure 3:**
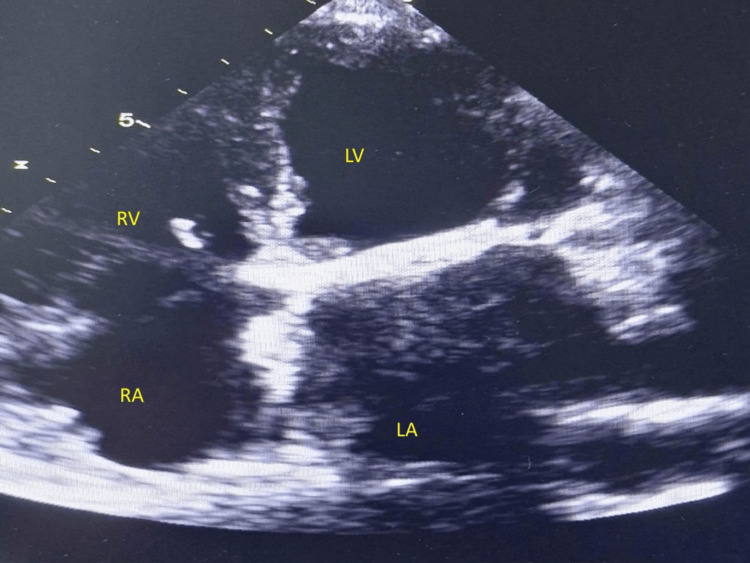
Echocardiography after thrombolysis demonstrating thrombus resolution in four chamber apical view (4C) during first presentation 4C - apical four chamber view, LA - left atrium; LV - left ventricle; RA - right atrium; RV - right ventricle

**Figure 4:**
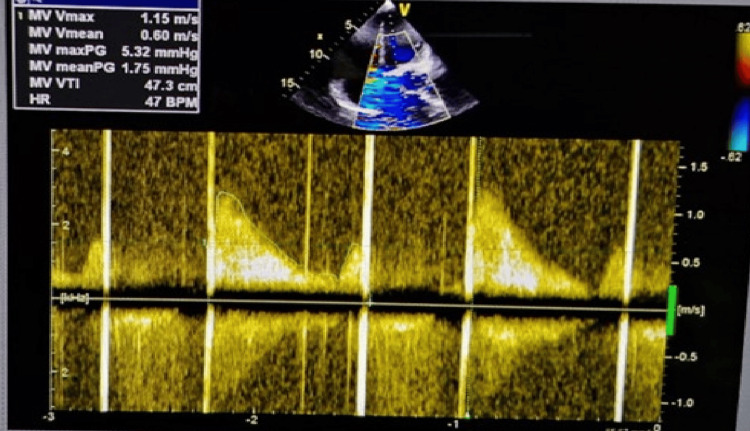
Post-thrombolysis Doppler ultrasound during first presentation Post-thrombolysis Doppler showing normalization of transmitral gradients (Pmax 5.32 mmHg, Pmean 1.75 mmHg), confirming non-obstructed mitral orifice. Pmax - peak gradient; Pmean - mean gradient

The clinical response was favorable. Within 24 hours of thrombolysis initiation, the patient experienced significant symptomatic improvement with reduced dyspnea and improved oxygen saturation (96% on 2 liters nasal cannula). By 48 hours, he demonstrated marked clinical improvement with dyspnea reduced to NYHA class II, improved exercise tolerance, and resolution of orthopnea. Repeat transthoracic echocardiography performed 48 hours post-thrombolysis demonstrated substantial improvement with a reduction of mean transmitral gradient to 1.75 mmHg and improved leaflet motion (Figures 3-4). 

No thromboembolic complications occurred during hospitalization, including absence of stroke, transient ischemic attack, or peripheral embolization. No major bleeding events were observed. The patient's neurological examination remained normal throughout the hospitalization. He was transitioned from heparin to therapeutic warfarin anticoagulation, achieving a therapeutic INR of 2.7 prior to discharge on day 5, in stable haemodynamic condition with detailed anticoagulation education provided to both patient and family members, emphasizing the critical importance of consistent warfarin use, regular INR monitoring, dietary considerations, and early recognition of warning symptoms requiring urgent medical attention.

Interval course between episodes

Following discharge, the patient remained clinically stable (NYHA functional class I-II) for several months with acceptable functional capacity and quality of life. However, despite intensive education during the first admission, socioeconomic constraints continued to significantly limit his access to regular INR monitoring and regular follow-ups in a timely manner. Rural geographic remoteness created logistical challenges for frequent laboratory visits. Consequently, multiple documented subtherapeutic INR values were recorded during sporadic follow-up visits, ranging from 1.7 to 2.4, indicating persistent inadequate anticoagulation - a concerning pattern that placed him at high risk for recurrent thrombosis.

Second episode of prosthetic valve thrombosis in 2025

Approximately two years after the first episode, in early 2025, the patient presented to the emergency department with a three-day history of rapidly worsening dyspnea, orthopnea, progressive fatigue, and reduced exercise tolerance. The clinical presentation was strikingly similar to his first episode. On detailed questioning, he admitted to missing several consecutive warfarin doses over the preceding four weeks and acknowledged non-monitoring of his INR for several months.

On arrival, physical examination revealed a patient in moderate-to-severe respiratory distress with tachypnea (respiratory rate 28 breaths/minute), tachycardia (heart rate 115 beats/minute), blood pressure 90/60 mmHg, and oxygen saturation 90% on room air. Cardiovascular examination revealed diminished mechanical mitral valve closing click, an apical holosystolic pansystolic murmur radiating to the axilla, and elevated jugular venous pressure. Respiratory examination demonstrated bilateral pulmonary crackles extending to mid-lung zones bilaterally. Mild bilateral lower extremity edema was present.

Laboratory testing confirmed subtherapeutic anticoagulation with INR values of 1.7. Other laboratory parameters revealed CK-MB of 12 U/L (normal range: 0-25 U/L) and troponin-I of 0.006 ng/mL (normal range: <0.04 ng/mL), ruling out acute myocardial ischemia. The complete blood count was within normal limits, including a hemoglobin of 13.8 g/dL (normal range: 13.5-17.5 g/dL), white blood cell count of 6.1 ×10⁹/L (normal range: 4.0-11.0 ×10⁹/L), and platelet count of 234 ×10⁹/L (normal range: 150-450 ×10⁹/L). Renal function tests were unremarkable, with a serum creatinine of 0.95 mg/dL (normal range: 0.6-1.3 mg/dL) and blood urea nitrogen of 15 mg/dL (normal range: 7-20 mg/dL). Liver function tests were also normal, including AST 30 U/L (normal range: 10-40 U/L), ALT 48 U/L (normal range: 7-56 U/L), and total bilirubin 0.9 mg/dL (normal range: 0.2-1.2 mg/dL). Inflammatory markers were within normal limits, with an ESR of 12 mm/hr (normal range: <20 mm/hr) and a CRP of 2.4 mg/L (normal range: <5 mg/L). Serum electrolytes were also normal, including sodium 142 mmol/L (normal range: 135-145 mmol/L), potassium 4.8 mmol/L (normal range: 3.5-5.0 mmol/L), and chloride 104 mmol/L (normal range: 98-107 mmol/L). Electrocardiography demonstrated sinus tachycardia without acute ischemic changes. Chest radiography showed cardiomegaly with prominent pulmonary vascular markings.

**Figure 5:**
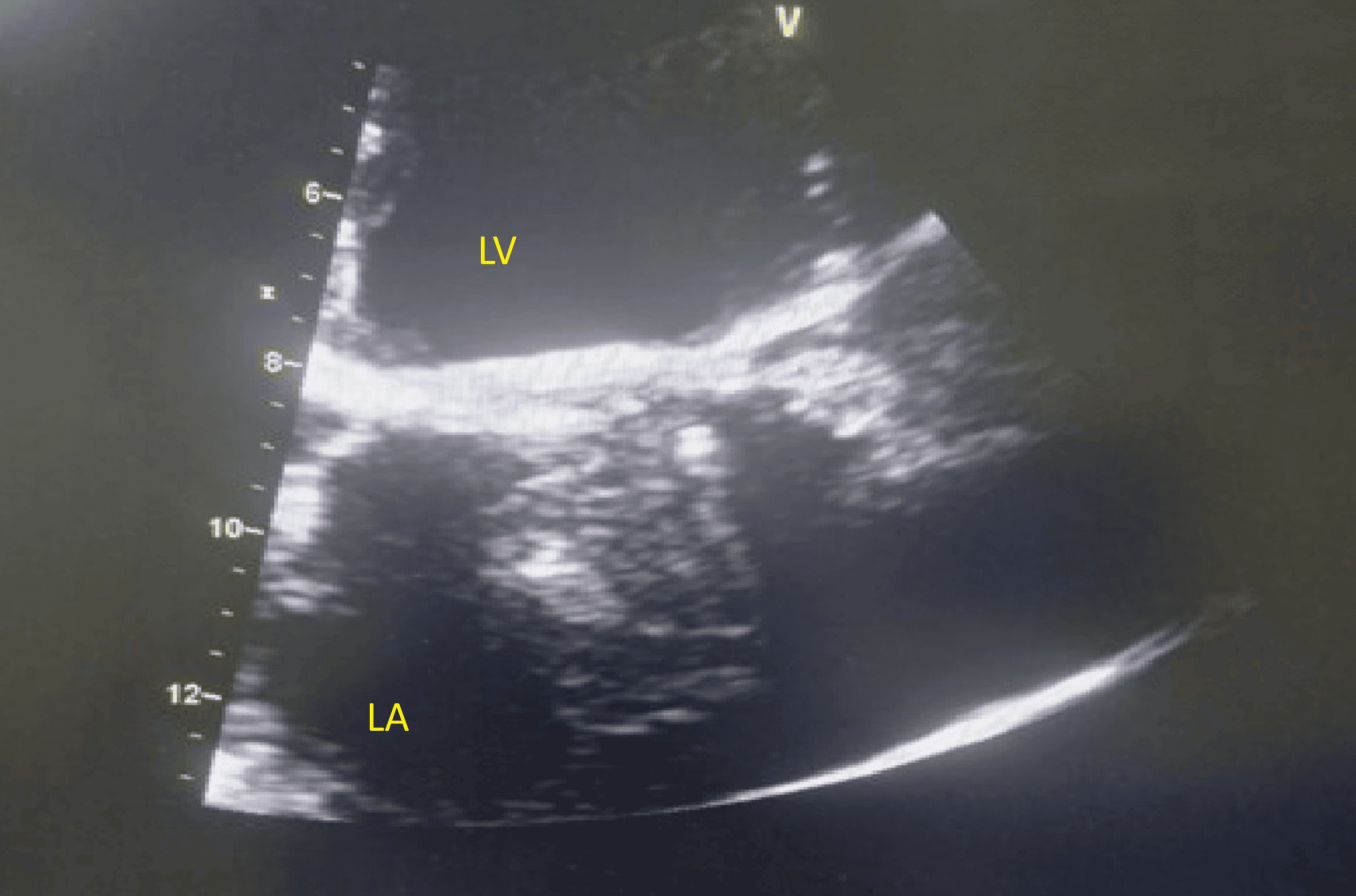
Echocardiography of the second episode Echocardiography of the second episode showing recurrent obstructive thrombus on the mitral prosthesis in a zoomed four-chamber apical view (4C), along with reverberation artifact due to the mechanical mitral prosthesis. LA - left atrium; LV - left ventricle

**Figure 6:**
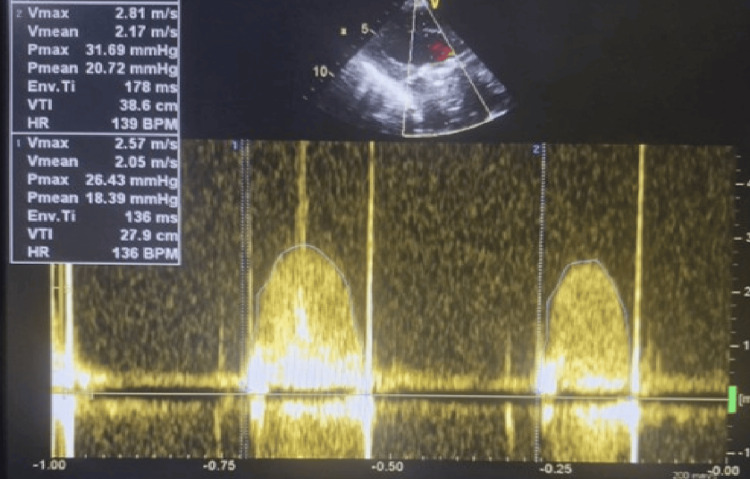
Echocardiography with continuous wave Doppler ultrasound Echocardiography with continuous wave Doppler demonstrating recurrent elevation of transmitral gradients (Pmax1 26.43 mmHg, Pmean1 18.39 mmHg, Pmax2 31.69 mmHg, Pmean2 20.72mmHg), confirming recurrent prosthetic valve obstruction. Pmax - peak gradient; Pmean - mean gradient

Transthoracic echocardiography demonstrated findings consistent with recurrent obstructive PVT: significantly elevated gradients (average peak gradient 29 mmHg, average mean gradient 19.5 mmHg), markedly restricted prosthetic leaflet excursion, and visible echodensities on the valve leaflets, measuring approximately 1.0×0.7 cm (Figures 5-6), adherent to the atrial surface of the mechanical valve. Left ventricular systolic function remained preserved with an ejection fraction 56%.

The multidisciplinary team reconvened urgently to discuss management options. Factors considered included the patient's previous highly successful response to thrombolytic therapy without complications, continued absence of readily available surgical intervention with similar or greater delays anticipated, absence of contraindications to thrombolysis (no recent surgery, bleeding, or stroke), and the patient's strong preference for medical management based on his previous positive experience and ongoing financial constraints. After comprehensive shared decision-making involving the patient, family members, and medical team, the decision was made to proceed with a course of thrombolytic therapy using the identical protocol that had proven successful previously.

Second thrombolysis course and outcome

The patient was admitted to the cardiac intensive care unit with the same comprehensive monitoring protocols established during the first episode. The identical low-dose, slow-infusion streptokinase protocol was administered: 250,000 IU loading dose over 30 minutes followed by 100,000 IU/hour continuous infusion for 24 hours. Despite concerns about potential antibody formation from prior streptokinase exposure reducing efficacy, the nearly two-year interval between administrations suggested sufficient time for antibody levels to decline, and streptokinase was selected again based on cost considerations and local availability. Intensive monitoring included continuous cardiac telemetry, pulse oximetry, hourly vital signs, serial neurological assessments every two hours, and careful surveillance for bleeding complications.

**Figure 7:**
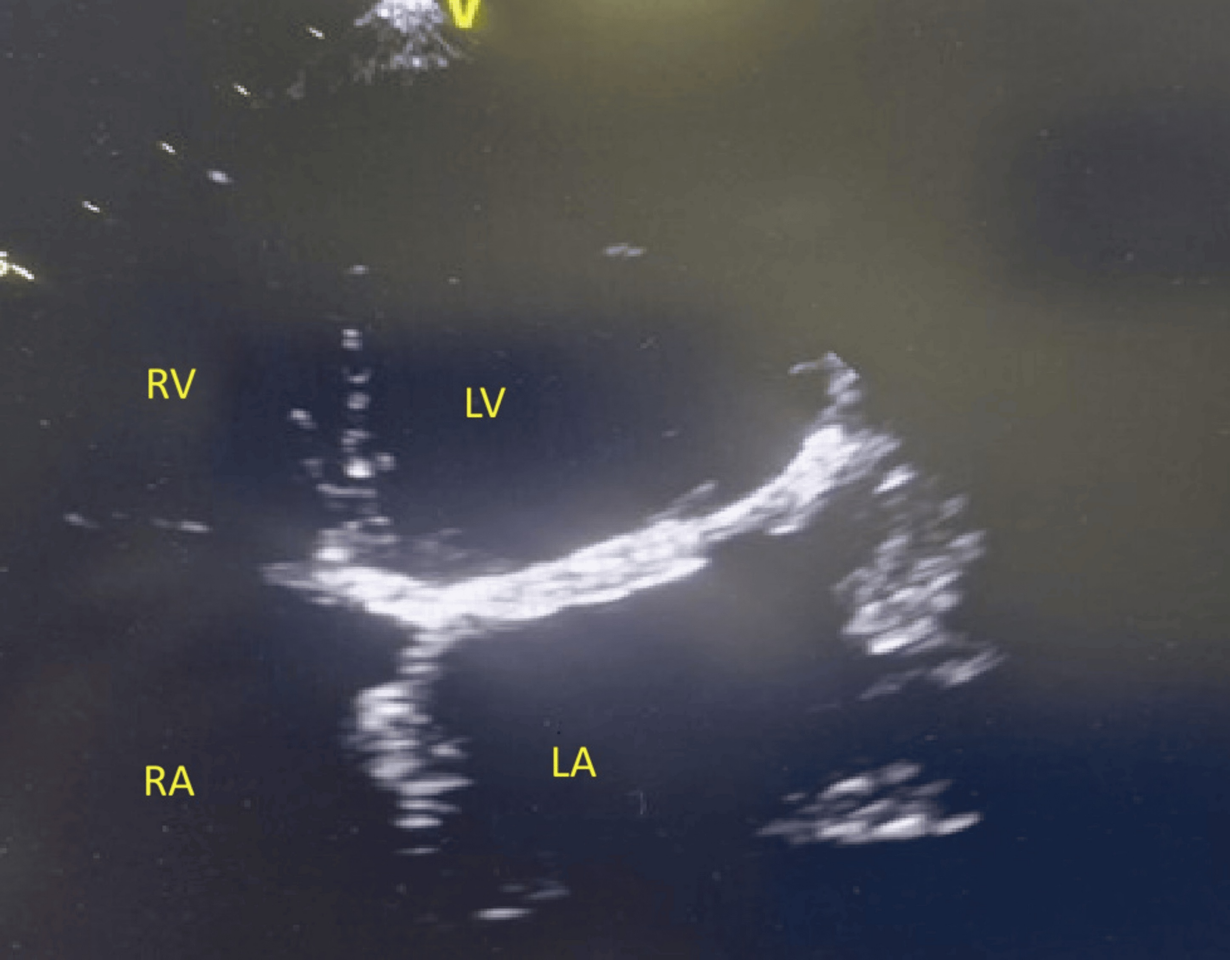
Echocardiography after second thrombolysis showing resolution of thrombus in apical four chamber view (4C) LA - left atrium; LV - left ventricle; RA - right atrium; RV - right ventricle

**Figure 8:**
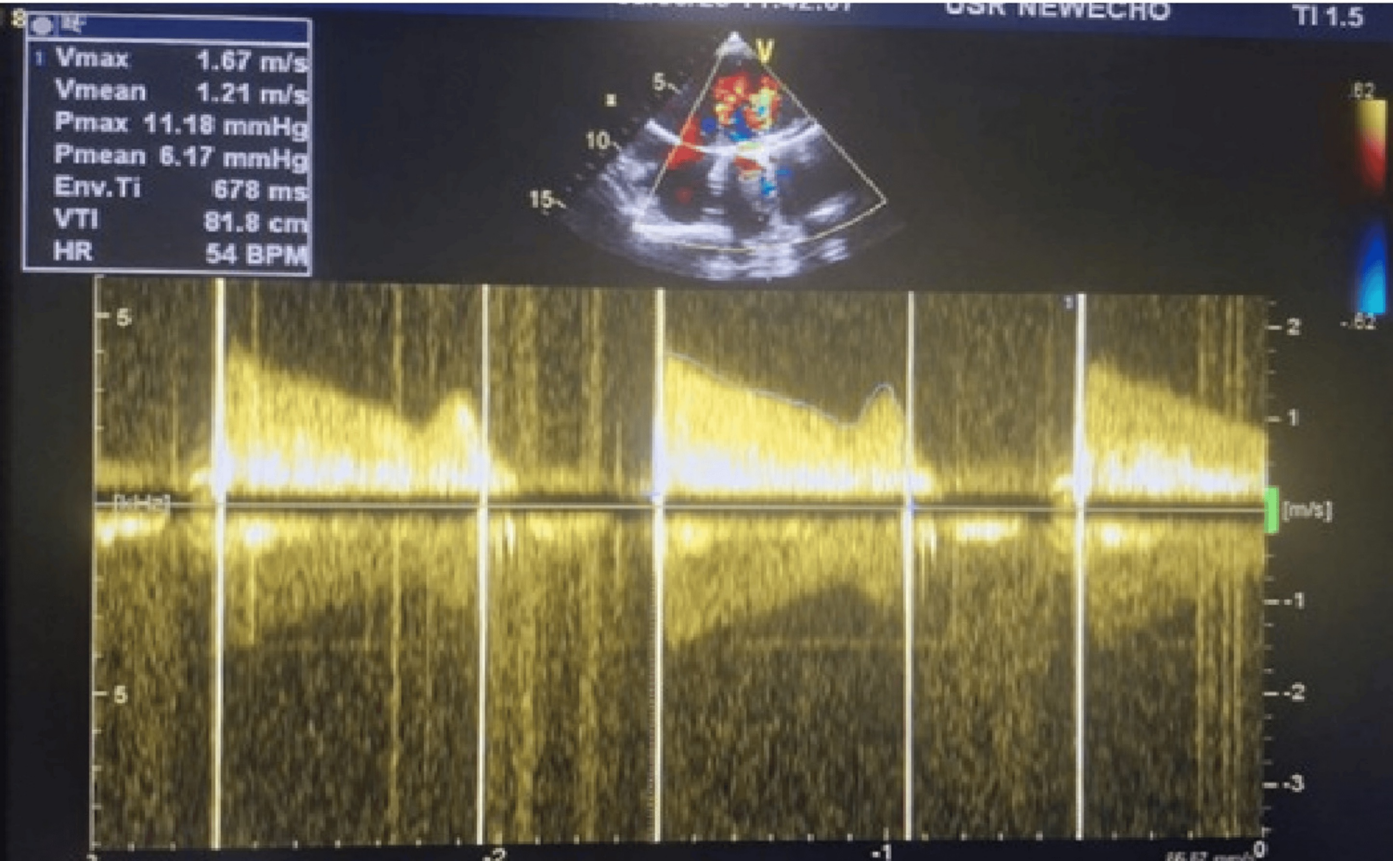
Echocardiography of the second episode of post-thrombolysis with continuous wave Doppler ultrasound Echocardiography of the second episode of post-thrombolysis with continuous wave Doppler demonstrating successful normalization of transmitral gradients (Pmax 11.18 mmHg, Pmean 6.17 mmHg). Pmax - peak gradient; Pmean - mean gradient

The clinical course mirrored the first episode with excellent therapeutic response. Within 24 hours of thrombolysis initiation, symptomatic improvement was noted with reduced respiratory distress and improved oxygen saturation. Progressive resolution of dyspnea continued over 24-72 hours, with the patient achieving NYHA class II functional status by 48 hours. Serial transthoracic echocardiography demonstrated gradual but consistent improvement in valve hemodynamics, with mean gradient reduction to 6.2 mmHg at 48 hours (Figures 7-8).

Importantly, no complications occurred during the second admission. There were no thromboembolic events, including stroke, transient ischemic attacks, or peripheral embolization. No major bleeding complications were observed. Neurological examination remained completely normal throughout hospitalization without any focal deficits or changes in mental status. The patient successfully transitioned back to therapeutic warfarin anticoagulation, achieving an INR level of 3.2 at the time of discharge on day 6 in stable condition with NYHA class I functional status.

The comparison of clinical, laboratory, and echocardiographic parameters across two episodes of prosthetic valve thrombosis is outlined in Table 1. 

**Table 1:** Comparison of clinical, laboratory, and echocardiographic parameters across two episodes of prosthetic valve thrombosis INR - international normalized ratio; NYHA class - New York Heart Association functional classification of heart failure severity

Parameter	Episode 1 in 2023	Episode 2 in 2025
INR (at presentation)	1.3	1.7
NYHA class (at presentation)	IV	IV
Mean transmitral gradient (at presentation)	24 mmHg	19.5 mmHg
Peak transmitral gradient (at presentation)	35 mmHg	29-31 mmHg
Thrombus size (echo)	1.2×0.8 cm	1.0×0.7 cm
Post-thrombolysis mean gradient	1.75 mmHg	6.2 mmHg
Post-thrombolysis NYHA class	II	II
NYHA class at discharge	I	I

Given the recurrent nature of PVT in this patient and recognition of significant barriers to adequate anticoagulation management, more intensive patient and family education with involvement of multiple family members to provide redundancy in medication administration and monitoring support, provision of detailed written instructions in the patient's native language was implemented.

## Discussion

This case report provides valuable insights into the management of recurrent obstructive mechanical mitral valve thrombosis in a resource-limited setting, demonstrating the feasibility, safety, and reproducibility of repeated thrombolytic therapy as a life-saving alternative when surgical intervention is not readily accessible. Obstructive PVT remains a rare but life-threatening complication that continues to challenge clinicians worldwide, particularly in resource-constrained environments where access to emergency cardiac surgery is limited or significantly delayed [1,2,6,7].

Diagnostic approach and clinical presentation

Accurate and timely diagnosis of PVT requires a high index of clinical suspicion combined with appropriate multimodality imaging. The clinical presentation can range from subtle symptoms such as diminished valve clicks or mild dyspnea to dramatic manifestations including acute pulmonary edema, cardiogenic shock, or sudden cardiac death [2,3]. In this case, both presentations were characterized by the classic triad of symptoms: progressive dyspnea, diminished or absent mechanical valve clicks on auscultation, and documented subtherapeutic anticoagulation-all highly suggestive of obstructive PVT [2,8].

Transthoracic echocardiography serves as the initial imaging modality, offering assessment of transvalvular gradients, leaflet motion, and direct visualization of thrombus. However, transesophageal echocardiography provides superior diagnostic accuracy and is considered the gold standard for PVT diagnosis, offering detailed visualization of thrombus size, location, mobility, attachment site, and degree of valve obstruction-all critical parameters for therapeutic decision-making and risk stratification [2,5]. 

Treatment decisions in resource-limited settings

Current ESC/EACTS and ACC/AHA guidelines recommend urgent surgical intervention, typically redo valve replacement or thrombectomy, as first-line therapy for obstructive left-sided PVT, particularly for critically ill patients with NYHA class III-IV symptoms [1,3,4]. However, these guideline recommendations are based primarily on data from well-resourced healthcare systems with readily available cardiac surgical services and may not be directly applicable or feasible in many resource-limited settings worldwide [6,7].

Real-world management in low-resource environments is frequently constrained by multiple factors including absence of on-site cardiac surgical facilities, limited availability of qualified cardiothoracic surgeons with experience in reoperative valve surgery, lack of comprehensive perioperative cardiac critical care infrastructure necessary for reoperations, significant delays in arranging urgent surgical intervention, patient transfer, and substantial financial barriers to surgery and postoperative care for many patients [6,7]. In such contexts, thrombolytic therapy may represent not merely an alternative treatment option but potentially the only feasible life-saving intervention for critically ill patients with obstructive PVT.

In this report, successful thrombolysis was defined by a combination of clinical improvement (resolution of heart failure symptoms), marked reduction of transvalvular gradients with restored leaflet motion on TTE, and absence of major complications such as stroke, systemic embolization, or major bleeding.

In this case, the decision to proceed with thrombolytic therapy in both episodes was driven by multiple converging factors: absence of accessible emergency cardiac surgical facilities within a reasonable timeframe, patient's critical hemodynamic status requiring immediate intervention, absence of contraindications to thrombolytic therapy, patient and family preference after comprehensive counseling, and for the second episode, the previous highly successful outcome with thrombolysis without complications [9,11,14].

Thrombolytic protocol and agent selection

Multiple thrombolytic agents and protocols have been utilized for PVT management with varying success rates and safety profiles. Streptokinase, the agent employed in this case, offers several practical advantages, particularly relevant to resource-limited settings: significantly lower cost compared to newer thrombolytic agents such as rtPA or tenecteplase, widespread availability in developing countries, extensive clinical experience, and documented efficacy in PVT with success rates comparable to more expensive alternatives [8,11-15].

The low-dose, slow-infusion protocol utilized in both episodes (250,000 IU loading dose followed by 100,000 IU/hour for 24 hours) represents an evolution in thrombolytic strategies for PVT. Earlier protocols employed higher doses administered more rapidly; however, accumulating evidence suggests that slower infusion rates over prolonged periods may achieve similar efficacy while reducing the risk of major complications, particularly systemic embolization and major bleeding [11,12,15]. The rationale for slow infusion is that gradual thrombus dissolution reduces the likelihood of large embolic fragments breaking free while allowing time for endogenous hemostatic mechanisms to stabilize.

Repeated thrombolysis: safety and efficacy

The successful use of repeated thrombolytic therapy in this case is particularly noteworthy, as the medical literature contains relatively few reports of successful repeated thrombolysis for recurrent PVT episodes in the same patient [14,18]. Most published case series and cohort studies describe single episodes of PVT treated with thrombolysis, and there are valid concerns about repeated thrombolytic administration, including potential antibody formation (particularly relevant for streptokinase, which is immunogenic), and cumulative risk of bleeding complications [8,11].

In this patient, several factors likely contributed to the successful repeat thrombolysis. The two episodes occurred more than two years apart, well beyond the typical six-month period during which streptokinase antibodies persist at potentially neutralizing levels, suggesting that antibody-mediated resistance was unlikely [8]. Each episode was clearly and directly attributable to documented subtherapeutic anticoagulation rather than structural valve problems, pannus formation, or other pathology that might limit thrombolytic efficacy [2]. The patient's overall good health status with preserved ventricular function and absence of major comorbidities provided a favorable substrate for successful treatment. The comprehensive monitoring protocols and intensive supportive care in a well-equipped ICU setting further minimized complication risk [11,12].

The favorable outcomes achieved in both episodes, with complete thrombus resolution, restoration of normal valve function, and absence of major complications including stroke, major bleeding, or death, provide important evidence supporting the feasibility of repeated thrombolysis in appropriately selected patients when surgical alternatives are not available [9,14,18].

Outcomes and complications

The success rates for thrombolytic therapy in PVT reported in the literature vary widely, ranging from 60% to 90% depending on patient selection criteria, thrombus characteristics, thrombolytic agent and protocol used, and definition of success [6,7,11-15]. The outcomes in this case-rapid symptomatic improvement within 24-48 hours, thrombus resolution on TTE, normalization of valve hemodynamics with mean gradients returning to acceptable ranges for mechanical mitral prosthesis, and short hospital stays-align with the favorable end of reported outcomes for thrombolytic therapy in PVT [11,12,15].

Critically important is the absence of major complications in both treatment courses. Thrombolytic therapy for PVT carries well-recognized risks, with systemic embolization and stroke representing the most feared complications occurring in approximately 10-20% of cases in various series, and major bleeding complications occurring in 3-8% of patients [9,16,17]. Several factors may have contributed to the favorable safety profile in this patient, including careful patient selection, use of the low-dose, slow-infusion protocol, which may reduce embolic risk, intensive monitoring in an ICU setting, allowing early detection and management of potential complications, and absence of additional bleeding risk factors or contraindications [11,12,15].

The challenge of anticoagulation management and recurrence prevention

The recurrence of PVT in this patient despite successful initial treatment and education highlights one of the most significant challenges in managing patients with mechanical heart valves in resource-limited settings: ensuring adequate long-term anticoagulation. Subtherapeutic anticoagulation remains the most important modifiable risk factor for PVT, identified in up to 70-80% of cases [2,6,7]. In low- and middle-income countries, multiple barriers contribute to inadequate anticoagulation control including financial constraints limiting access to warfarin and INR monitoring, geographic remoteness creating logistical challenges for regular laboratory visits, limited availability of dedicated anticoagulation clinics and specialized services, inadequate health literacy affecting understanding of anticoagulation importance, cultural factors and health beliefs that may influence adherence, and competing priorities for limited financial resources in economically disadvantaged populations [6,7].

Effective prevention of recurrent PVT requires comprehensive, multifaceted interventions addressing these barriers. Evidence-based strategies include intensive patient and family education programs emphasizing the critical importance of consistent anticoagulation, recognition of warning signs, and medication adherence; establishment of subsidized or free access programs for warfarin and INR monitoring to address financial barriers; development of community-based anticoagulation clinics bringing services closer to patients' residences; implementation of point-of-care INR testing devices that can be used in rural or remote settings; use of telephone and mobile health technologies for monitoring support and medication reminders; family involvement in medication administration and monitoring to provide redundancy; and systematic follow-up protocols with regular clinic visits and proactive outreach for missed appointments [6,7].

Direct oral anticoagulants (DOACs), which offer potential advantages of fixed dosing without monitoring, are unfortunately not appropriate alternatives for patients with mechanical heart valves due to demonstrated lack of efficacy and increased risk of complications in this population [10]. Therefore, optimizing warfarin management remains essential for preventing PVT recurrence.

Guideline implications and adaptation to local context

This case raises important questions about guideline applicability and adaptation to resource-limited contexts. While international guidelines appropriately recommend surgery as first-line therapy for obstructive left-sided PVT based on evidence from high-resource settings [1,3,4], the feasibility of implementing these recommendations varies dramatically across different healthcare systems globally. A growing body of evidence, including the current case, suggests that thrombolytic therapy can provide excellent outcomes when applied with appropriate protocols, careful patient selection, and comprehensive monitoring in settings where surgery is unavailable or significantly delayed [5,11-15,17,18].

There may be value in developing context-adapted guidelines or clinical pathways that explicitly address management of PVT in resource-limited settings, providing evidence-based recommendations for patient selection for thrombolysis, optimal thrombolytic protocols balancing efficacy and safety, comprehensive monitoring strategies, risk stratification and identification of patients at higher risk for complications, and systems-level interventions to improve access to both acute PVT management and preventive anticoagulation services. Such adapted guidelines could help clinicians in resource-constrained environments make evidence-informed decisions while acknowledging local constraints and resources [9,16].

Limitations

This case report has several important limitations that warrant acknowledgment. First, transesophageal echocardiography and fluoroscopic assessment were not performed at the time of presentation. Although TEE is considered the gold standard for detailed characterization of prosthetic valve thrombosis, it was deferred during both episodes due to the patient's critical clinical status and the urgent need for immediate life-saving intervention. Moreover, the patient demonstrated rapid clinical and transthoracic echocardiographic improvement following thrombolytic therapy, which precluded any subsequent opportunity to perform TEE. Management decisions were therefore guided by concordant clinical findings and unequivocal transthoracic echocardiographic evidence of obstructive prosthetic valve thrombosis. As a single-patient case report, the generalizability of these findings to broader patient populations is inherently limited, and systematic comparative data are unavailable. Additionally, the relatively short follow-up period after the second episode limits assessment of long-term outcomes and recurrence risk beyond the intermediate term. The inability to obtain cardiac surgical consultation during either episode further restricts evaluation of potential surgical feasibility and outcomes, although logistical and financial constraints suggest that surgical management would have been extremely challenging. Finally, resource limitations prevented a comprehensive thrombophilia workup, which may have identified additional underlying prothrombotic risk factors beyond subtherapeutic anticoagulation.

## Conclusions

This case demonstrates that repeated thrombolytic therapy can be a feasible and effective treatment for recurrent obstructive mechanical mitral valve thrombosis when surgical intervention is unavailable. Successful resolution of valve obstruction on two separate occasions without major complications highlights the potential role of thrombolysis in carefully selected patients under close clinical and echocardiographic monitoring. The case also emphasizes the importance of sustained anticoagulation support and patient education to reduce the risk of recurrence, particularly in settings where access to definitive surgical care is limited.
